# Supplementary material for: Effect of Length of Cellulose Nanofibers on Mechanical Reinforcement of Polyvinyl Alcohol
Source: Polymers (Basel). 2021 Dec 30;14(1):128. doi: 10.3390/polym14010128 (PMC8747125; doi:10.3390/polym14010128)
Supplement: Supplementary file 1 [file polymers-14-00128-s001.zip › polymers-1488970-supplementary.pdf]

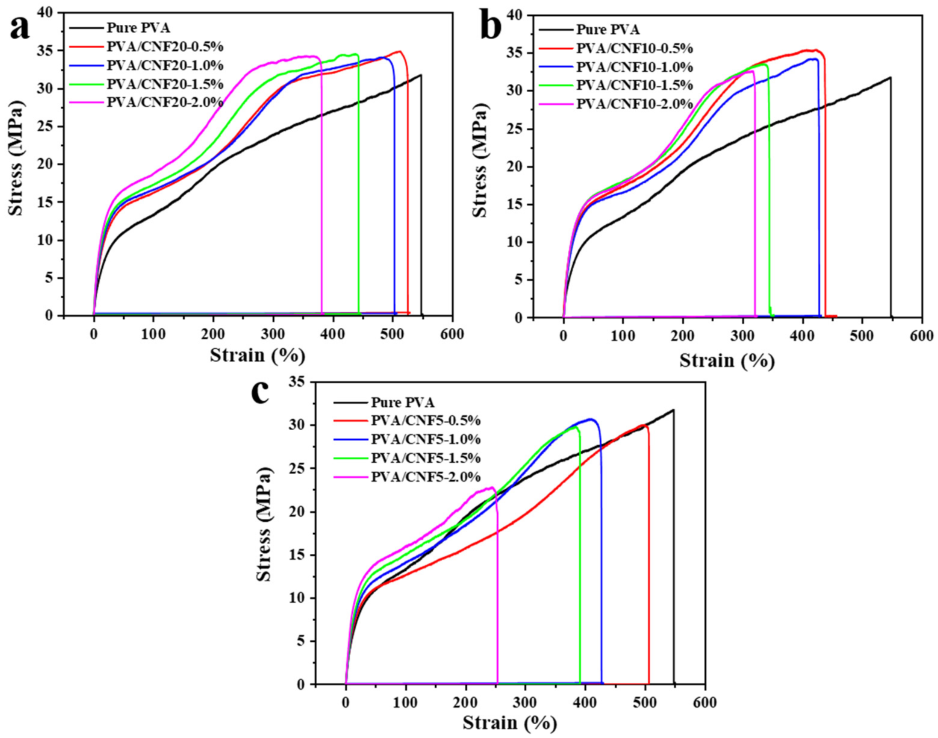


**Figure S1**. Stress-Strain curves of pure PVA and PVA/CNF composites with various concentration of CNF.
